# All That We Need to Know About the Current and Past Outbreaks of Monkeypox: A Narrative Review

**DOI:** 10.7759/cureus.31109

**Published:** 2022-11-04

**Authors:** Shruti Singh, Rajesh Kumar, Sunil K Singh

**Affiliations:** 1 Pharmacology, All India Institute of Medical Sciences, Patna, Patna, IND

**Keywords:** zoonotic virus, infectious disease, men who have sex with other men (msm), monkeypox vaccines, smallpox vaccine, tecovirimat, monkeypox

## Abstract

Monkeypox is a rare, zoonotic viral illness that was initially endemic mainly to Africa. The virus later spread to non-African countries and, in 2022, it exploded on a global scale, with an unprecedented number of cases. The rapid, multi-country transmission, primarily in men who have sex with men (MSM), and evolving clinical manifestations and demographic traits of the current outbreak have raised serious concerns among the international health community. An in-depth inquiry into the past and present outbreaks are required, especially when contrasting features are witnessed across countries and outbreaks. This narrative review aims to summarize the evolution in the epidemiology, clinical features, mode of transmission, and management protocol of human monkeypox infection (H-MPVX) over the decades. For a detailed characterization of the novelties associated with the current outbreak that would facilitate the accurate dissemination of information and policy-making, we performed a thorough literature search for MPVX infection on PubMed, Google Scholar, and Science Direct, using appropriate keywords to choose relevant articles.

## Introduction and background

Monkeypox is a zoonotic disease caused by the monkeypox virus (MPXV). Over the last few decades, it has been an epidemic infection among African countries. However, in 2003 it was witnessed out of Africa in the United States for the first time. In 2022, MPXV infection spread worldwide. There is great concern about the global spread of MPVX and changing disease course [1,2].

There are several doubts regarding the disease spectrum, transmission modes, risk factors for hospitalization, and prevention and management of the human MPVX (H-MPVX). The recent outbreak has added to the fear of the spread of MPVX infection worldwide like the spread of coronavirus disease 2019 (COVID-19) in the population. Several research papers have, therefore, been published recently to analyze the evolving traits [1-5].

Here, we have attempted to provide a comprehensive review of the virus etymology, historical background, clinical picture, diagnosis, and management of MPVX based on the evidence generated from past and current epidemiological studies. This review will be useful to improve the understanding of MPVX evolution, management, and prevention.

## Review

Family Poxviridae

The Poxviridaea family (poxviruses) consists of DNA viruses. These include the genera* Orthopoxvirus*, *Parapoxvirus,* *Avipoxvirus*, *Capripoxvirus*, *Leporipoxvirus*, *Suipoxvirus*, *Molluscipoxvirus*, and *Yatapoxvirus*. They all share a DNA sequence with similar antigens that demonstrate cross-reactivity [6]. The poxviruses that can affect humans include human smallpox (SPX) (variola virus), declared eradicated by the World Health Organization (WHO) in 1980, cowpox, used in making the SPX vaccine (vaccinia), MPVX, the most prevalent among humans, and few others (Table 1).

**Table 1:** Poxviruses that can affect humans with host and disease

Genera	Virus	Host	Disease
Molluscipoxvirus	Molluscum contagiosum	Humans	Multiple long-lasting skin nodules
Orthopoxvirus	Variola	Humans	Smallpox
	Vaccinia	Humans	Vesicular vaccination lesions
	Cowpox	Cattle, cats, rodents	Lesions on hands
	Monkeypox	Monkey, squirrels	Lesions resemble smallpox
Parapoxvirus	Pseudocowpox	Cattle	Mikers’ nodes
	Orf	Sheep, goats	Vesiculo-granulomatous lesions in localized areas
Yatapoxvirus	Tanapox	Monkeys	Fever and vesicles
	Yabapox	Monkeys	Local skin tumours

**Figure 1:**
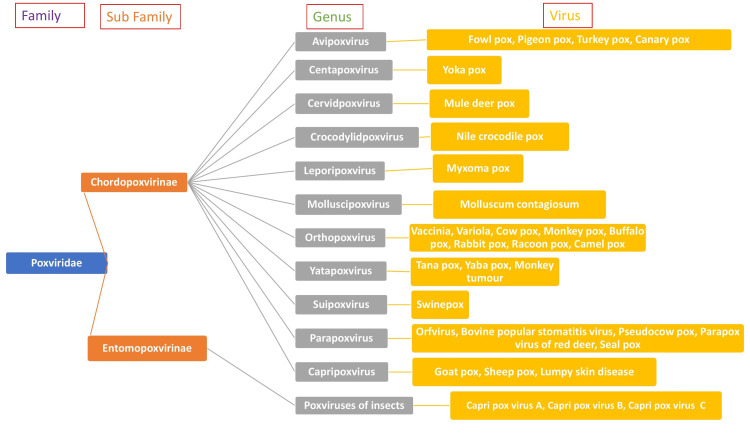
Classification of Poxviruses Image credit: Authors

Bovine SPX, with a significant reservoir in the animal kingdom, can also cause papulovesicular disease in humans but of less severity and generally very little mortality. The parapoxviruses are orf viruses that are widely disseminated among sheep, goats, and other cattle and produce milkers’ nodules in humans. These confer lesser immunity than orthopoxviruses, and reinfections are common. The Molluscipoxvirus causes molluscum contagiosum in humans, characterized by lesions resembling soft tumors in various body parts, especially the trunk and root of extremities. Molluscum contagiosum is spread by sexual and direct contact and also by fomites. The yatapoxviruses cause a vesicular-papular disease in exposed areas, an adenopathy reaction, and systematic symptoms for almost six weeks. Poxviruses do not have as much ability to mutate as other RNA viruses, such as severe acute respiratory syndrome coronavirus 2 (SARS-CoV-2). However, their virulence and ability to escape the immune system are noteworthy [7,8]. Orthopoxviruses, which are wreaking havoc around the globe and are currently a matter of public debate, include SPX and MPVX (Figure 1).

SPX and the development of tecovirimat

The death toll ascribed to SPX in the 20th century alone was 300 million. SPX had a mortality rate ranging from 10-75% [9-11]. History has witnessed massive epidemics of SPX periodically [12]. The incubation period for SPX was 7-14 days. A diffuse scarlatinian or macular rash followed, involving the skin mainly over the extremities and face initially and later on the trunk when it evolved into papules. About five or six days after their appearance, these papules changed into round vesicles with central umbilication, and around the eighth to the ninth day, clear pustules with greyish and turgid contents were seen. The crusts adhered to the skin for a long time and left scars when they fell off. Fortunately, this deadly disease was eradicated in 1980 due to extensive vaccination programs during the 1960s and 1970s, supplemented in the later stages by intense surveillance to isolate cases and ring vaccination of contacts and the fact that the only reservoir was humans [13-17].

However, the SPX virus can still be used as an agent of bioterrorism because even though the variola virus does not exist in nature, there are possibilities of clandestine stocks and re-emergence from natural sources [18]. Additionally, orthopoxviruses and poxviruses can be modified and created in laboratories with the help of molecular biology tools [18]. Moreover, the variola virus's legal stocks are also in the United States and Russian federations at WHO-approved repositories. Of particular note is today's absence of "herd immunity" in an unprotected population. Mass vaccination has been completely stopped due to the eradication of SPX and the excessive costs and significant side effects associated with vaccination. These are the reasons why SPX is of particular interest, especially in the context of biological warfare [19]. Consequently, significant funds are invested in stockpiling medical countermeasures (MCM) vaccines and antivirals to improve the impact of an SPX epidemic.

The Public Health Emergency Medical Countermeasures Enterprise (PHEMCE) has been developed for targeted drug, vaccine, and device development against SPX and other health security threats [20]. As expected, routine clinical trials to check the efficacy and toxicity of the MCMs are impossible because the diseases are rare, cases are infrequent, and exposing volunteers to the pathogens is unethical. The FDA established the Animal Rule (Approval of New Drugs When Human Efficacy Studies Are Not Ethical or Feasible) to solve this problem. This rule permits sponsors to show a product's efficacy in animal models instead of a human efficacy trial. The rest of the aspects of a trial, such as the demonstration of safety (in Phase 3 on healthy adults) and Good Manufacturing Practices (GMP), remain the same [21,22]. To identify molecules with anti-poxvirus activity, libraries of compounds (consisting of already licensed molecules and novel chemical entities) were checked using high throughput screening [22]. Of the 300,000 compounds screened, nine chemical scaffolds demonstrated desirable activity, and finally, the lead compound ST-246, a 4-trifluoromethyl phenol derivative or TPOXX, was developed [22]. Tecovirimat (sold under brand name TPOXX) is a promising antiviral agent that exhibits potent anti-poxvirus activity and a low level of cytotoxicity. The animal models that best fulfilled all the criteria laid down under the Animal Rule to establish the antiviral efficacy of a molecule were the IV challenge of cynomolgus macaques with MPVX, the rabbitpox virus intradermal challenge model in rabbits, and the intranasal challenge model of ectromelia virus (mousepox) in Balb/C mice [23-27]. Subsequent trials in healthy human volunteers to ascertain toxicity, pharmacokinetics (PK) and pharmacodynamics (PD) parameters, human dose equivalents, and a pivotal clinical trial in 449 adults led to the approval of TPOXX on July 13, 2018, for SPX [28-32].

Many animals are also susceptible to infection with MPVX, including squirrels, dormice, rodents, and non-human primates (NHPs). Some of these animals have been used to understand poxvirus acquisition and transmission and to test protective drugs and vaccines [33,34].

MPVX: historical background

MPVX was first identified in captive cynomolgus monkeys (*Macaca fascicularis*) in Denmark during research for the poliovirus vaccine. There is confusion as to the true origins of the virus since these monkeys had been imported from Singapore and not Africa [35].

**Figure 2:**
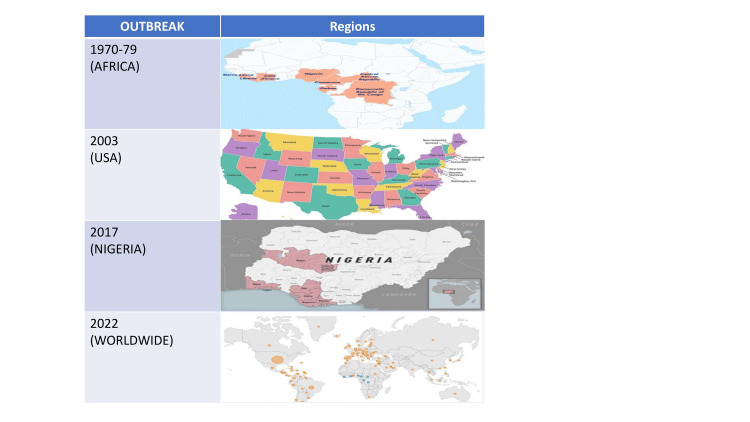
The pattern of monkeypox spread in the past and the current outbreak Image credit: Authors; Maps from CDC websites

It was believed that MPVX only affects animals for a long time, but the first human case was identified in a nine-month-old baby boy in Zaire in 1970 [36]. Later, outbreaks in 11 African countries, Cameroon, Benin, Democratic Republic of Congo (DRC), Gabon, Ivory Coast, Liberia, Sierra Leone Central African Republic, Nigeria, Republic of Congo, and South Sudan, occasionally occurred in humans who came in contact with animals (Figure 2).

**Figure 3:**
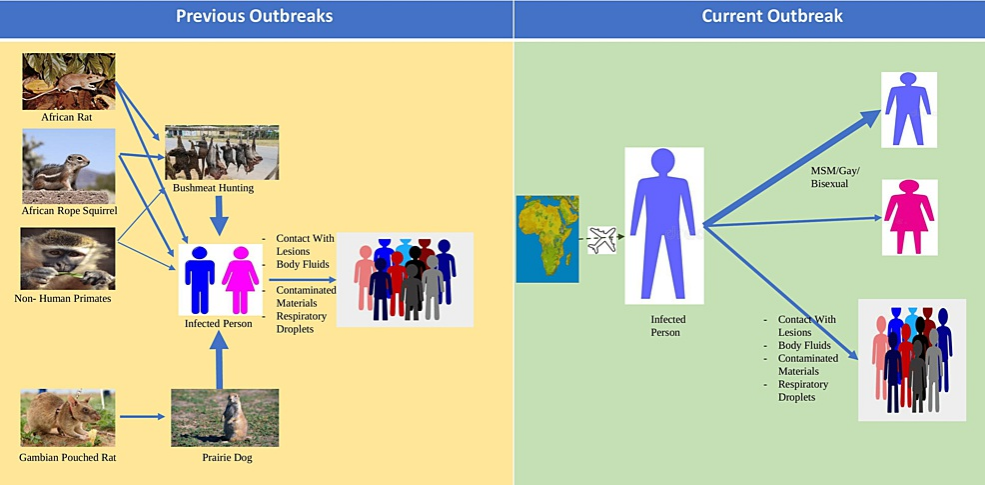
Changes in the transmission mode of monkeypox over the decades MSM: men who have sex with men Thin Arrow: low association; Thick Arrow: higher association Figure credit: Authors

The WHO reported in 1980 that there had been 47 human cases of MPVX infection since 1970 clustered around Central and West Africa; 38 of these cases were reported in the DRC [37]. Not much was known about the natural reservoir of the virus earlier; even now, we are uncertain about the natural reservoir of the virus. However, rodents like prairie dogs, squirrels, rabbits, and NHPs (considered accidental hosts), in whom *Orthopoxvirus* antibodies have been detected, might harbor the virus and cause disease [38-40]. For decades, MPVX was considered a zoonotic disease primarily, but the human-to-human transmission was witnessed in later outbreaks. However, even in these outbreaks, there was clear evidence of acquiring infection via a zoonotic transmission (Figure 3).

A research paper documented the signs and symptoms of H-MPVX patients in Congo from 1980-1985. Patients' ages ranged from one month to almost 70 years, and 90% were below 15 years of age. The crude mortality rate among unvaccinated was around 11%, and it was higher among children [41].

Cases continued to rise during the 1990s, with another large outbreak (511 confirmed, probable, and/or possible monkeypox cases) in 1996-1997 in the DRC and nine confirmed cases in Gabon [42,43]. This outbreak had a low fatality rate but a higher-than-average attack rate. The DRC has been the country most affected by MPVX, with continuous reports of H-MPVX cases over the last five decades. Just recently, in 2020, 4,594 suspected cases were reported from the DRC [44]. Nigeria has been the second most affected country, with 181 confirmed and probable cases from a September 2017 outbreak [45]. The Republic of Congo (n=97) and Central African Republic (n=69) have been the third and fourth most affected countries, respectively. All the rest of the African countries together contributed to less than a total of 20 cases over the last five decades. The United States outbreak of 2003 heralded the non-endemic spread of the virus. This outbreak affected 47 confirmed or probable cases in the United States in people who had come in contact with infected pet prairie dogs who had acquired the infection from exotic animals imported from Ghana [46,47]. Since then, several outbreaks of H-MPVX have occurred sporadically. The largest known outbreak happened in Nigeria in 2017, nearly 40 years after the previous case. There was one report from Singapore on a returning traveler from Nigeria in May 2019. Later three members of the same family who had traveled from Nigeria to the United Kingdom were confirmed infected in 2021. In 2021, another H-MPVX infection was identified in a man who had moved from Nigeria to Maryland, United States. Three H-MPVX cases were also reported in the United Kingdom between 2018 and 2019. The outbreak of 2022, just like previous outbreaks, began in the United Kingdom with a man who had traveled to Nigeria. Later, infection was also detected in two cohabitants of the first case [48-51] (Figure 2).

As of October 14, 2022, the CDC documented 72,547 cases in 109 locations. Out of these 109 locations, 102 are countries that have historically never reported H-MPVX. A total of 26 deaths have been reported from 12 locations. The number of cases reported in the first week of this outbreak has surpassed the total number of cases in Western and Central Africa in the last 40 years. The total number might be an underestimation, with the taboo associated with MPVX due to its prevalence in the men who have sex with other men (MSM) community preventing people from coming forward when they have symptoms. This trend is unlike past outbreaks, localized in Africa or linked to animal vectors or travel history to endemic regions. Most cases in the recent outbreak have occurred through human-to-human transmission and in people with high-risk behaviors and MSM [52]. The frequent presentation of H-MPVX cases with anogenital lesions suggests that transmission has occurred through close physical contact [53-56] (Figure 3).

MPVX: Clades and Genomic Surveillance

Two separate clades of MPVX have been identified: Congo Basin (more virulent and infective) and West Africa. The case fatality ratio (CFR) for the Central African clade was higher than that of the West African clade. The West African clade seems responsible for some of the cases in this outbreak [52]. Phylogenetic analysis of the MPVX-2022 strain suggests that it has the same lineage as the MPVX- 2018 strain. However, despite this similarity, it still has 46 new consensus and 24 non-synonymous mutations suggesting genomic evolution that might be a reference for further studies and clinical correlates [57]. Some studies have reported a gene loss that correlates well with human-to-human transmission [58].

MPVX: clinical manifestations and transmission routes

The clinical manifestations, incidence, attack rate, mode of transmission, risk factors, and demographic characteristics of H-MPVX cases across the various outbreaks have been unusual and constantly changing. This variability depends upon age, gender, route of transmission, sexual orientation, degree of contact, vaccination status, the extent of exposure to the virus, patients’ physiological reserve, and the presence of comorbidities.

The symptoms of MPVX infection are similar but lesser in intensity than SPX and are rarely fatal, and they appear to resemble a modified form of SPX. Lymphadenopathy, occurring in the early part of MPVX, is the most contrasting feature differentiating MPVX from chicken pox and SPX in humans, a feature that has remained consistent across outbreaks [59].

Monkeypox is a re-emerging disease with a secondary attack rate of 1-10% in unvaccinated contacts against SPX. Most of the cases of H-MPVX in the world have occurred in people who were unvaccinated against SPX. Epidemiological data from the DRC (1981-2013) and the United States (2003) have confirmed that most H-MPVX cases (80-96%) have occurred in unvaccinated individuals. The greatest number of vaccinated individuals (21%) were present in the United States outbreak [60-62]. A study of monkeypox cases and suspects from Central Africa reported a much lower disease attack rate in the vaccinated population. Most MPVX infections in Africa historically occurred in children without an SPX vaccination [52].

Whenever there are multiple viruses targeting the same host at the same time, it is the virus with the greater R0 (basic reproduction number) that usually establishes infection. SPX had the greater R0 until its eradication; therefore, no MPVX cases were heard until 1980. With the eradication of SPX in 1980, we became susceptible to MPVX infection, and it was around that time that H- MPVX cases started appearing. In 2016, just a year before the outbreak in Nigeria, with the help of statistical modeling tools, researchers could conclude that population-level immunity had decreased from 65.6% in 1970 to 2.2% with the cessation of smallpox vaccination [58,63]. Today, the *Orthopoxvirus* immunity is at a mere 10%, and the R0 of MPVX is between 1.2 to 2.4; enough for human transmission [64]. Patients vaccinated against SPX present a milder clinical picture and fewer fatalities than those not vaccinated. Varicella-like pleomorphism has been observed to a greater degree in those vaccinated.

The age of H-MPVX-affected patients ranges from two years to 69 years [65]. Studies have reported that the weighted median age of H- MPVX infection in Africa has increased from around five years (1970-1980) to around 10 years and 21 years in the 2000s and 2010s, respectively. MPVX infection was more common in the younger population earlier. However, the current outbreak shows an increase in the median age of infection [60]. SPX vaccination campaigns were at their peak during the 1970s. In the 1980s, these vaccination drives were stopped due to the eradication of SPX. So, in the 2000s, only people less than 20 years of age were susceptible to MPVX since the remaining were already vaccinated.

**Figure 4:**
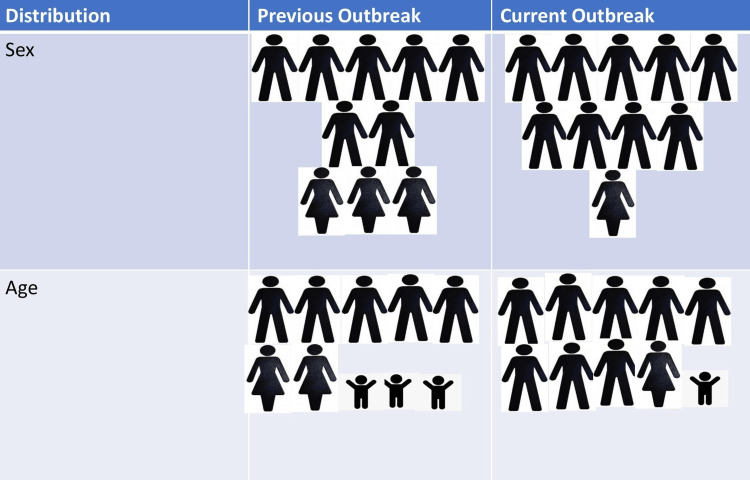
Distribution of monkeypox infection among population Image credit: Authors

The rise in the median age of infection in the current outbreak can be because of the disease affecting MSM predominantly. A similar justification may be offered for the greater male preponderance in the present outbreak. Although a meta-analysis of studies from 1980 to 2022 reported that males accounted for 57.6% of cases historically, mostly because traditionally, it is the men who have had more contact with wild animals, especially in endemic regions [66]. However, this level of significance was never before seen (Figure 4) [65].

Earlier, hardly any fatalities were reported in outbreaks occurring outside Africa. Almost 100% of deaths from the 1970s to 1990s were in children younger than 10 years of age. Between 2000 to 2019, only 37% of deaths were in children < 10 years. The Nigerian outbreak (2017-2018) reported a mean age of 27 years for the seven deaths in the 122 confirmed or probable cases of MPVX [60]. A 2022 meta-analysis by Benites-Zapata et al., which included publications from 1980 to 2022, reported a 4% mortality rate in hospitalized H-MPVX cases [65]. Overall mortality in the current outbreak has decreased compared to previous ones. The fact that children were affected more in the earlier outbreaks might contribute to this situation, along with a lack of antiviral drugs and vaccines. Bacterial superinfections, sepsis, and encephalitis have been reported more frequently among children and the immunocompromised. Even in high-income countries, these problems have escalated the mortality and hospitalization rate among children [67,68]. The CDC, therefore, started recommending that children with MPVX infection should ideally be admitted to a hospital or taken care of by a caregiver with a low risk of contracting the illness at home, and the antiviral drug tecovirimat should be started in children above the age of two years.

There was always a possibility that vulnerable groups would be affected more by increasing cases. However, few studies have analyzed the effect of MPVX infection in pregnant females. One landmark trial by Mbala et al. reported the outcomes of four pregnant women in a cohort of 222 symptomatic patients. Of these four women, only one (mild disease) gave birth to a healthy infant devoid of infection. Two women who had moderate and severe diseases, respectively, suffered miscarriages in the first trimester of pregnancy. Furthermore, one woman who was also coinfected with the malaria parasite delivered a stillborn, macerated fetus with characteristic MPVX lesions [69]. Another publication reported a premature infant being born to an infected pregnant mother. The infant also had an unmistakable rash suggestive of an MPVX infection. The infant died of unknown causes six weeks after its birth [70]. SPX infection data from historical papers also confirm that MPVX infection is more severe in pregnancy and might lead to fetal demise, especially in the third trimester [71,72]. Isolation and monitoring of the pregnant female and fetal ultrasound follow-up in symptomatic MPVX pregnant females to detect anomalies such as fetal hydrops and hepatomegaly early on are recommended. Tecovirimat and vaccinia immune globulin intravenous (VIGIV) might be considered in severely ill patients. The FDA has confirmed that no teratogenicity has been observed in animal studies with Tecovirimat. The CDC also permits vaccination with ACAM2000 in case of high-risk exposure to MPVX during pregnancy. However, patients might have to be counseled about the rare risk of fetal vaccinia that might lead to stillbirth, preterm delivery, and even death with ACAM2000 vaccination during pregnancy [73]. Needless to say, pregnant females are to be advised to stay as far away as possible from an H-MPVX case. As of now, not much is known about whether transmission can occur through breast milk. The WHO recommends covering the lesions and wearing a mask to decrease the chances of transmission. Nevertheless, this is an area where further research is required.

Traditionally, monkeypox has been recognized as a generalized, monomorphic, vesicular-pustular self-limiting disease that lasts three to four weeks [46]. The past outbreaks have offered crucial information about the epidemiology and clinical course of H-MPVX cases. It can take on the form of a systematic illness that can be mild to fatal. Signs and symptoms of MPVX usually appear 5-21 days after the infection and begin with a flu-like prodrome, including fever, fatigue, myalgia, chills, headache, and asthenia that evolve into a rash and lymphadenopathy (before or during inflammation). Lymphatic tissue involvement in H-MPVX cases might cause splenomegaly and hepatomegaly [74,75].

The most common signs of H-MPVX, according to a recent meta-analysis analyzing 1958 H-MPVX cases from 1980 to 2022, were rash (93%), pruritus (65%), lymphadenopathy (62%), fatigue (60%), sore throat (57%), headache (50%), cough (47%), myalgias (45%), photophobia (32%), arthralgia (26%), difficulty in breathing (25%), conjunctivitis (19%), nausea and vomiting (19%), and diarrhea (4%) [65].

Infections from most poxviruses have similar cutaneous manifestations. Centrifugal, well-circumscribed lesions (10 to 150) that progress from macules, papules, vesicles, and pustules concurrently and form crusted scabs over two to three weeks. The lesions may be filled with a white fluid, and they may later blister or turn into an abscess. Rarely hemorrhagic pustules might be seen.

The pustular stage may last for five to seven days before crust formation. During this time, a second febrile period with deteriorating symptoms might be seen. After one to two weeks, crusts evolve and desquamate. All types of Lesions may last for one to three days and evolve simultaneously. Skin lesions are mainly localized around the head, neck, and face (starting in the mouth) and extremities, with slightly lesser involvement of the trunk, hands, and soles of feet and very little involvement of legs, feet, pelvic area, groins, oral cavity, rectum, and genitalia. Cutaneous lesions are monomorphic rather than pleiomorphic and centrifugal rather than centripetal. MPVX cutaneous lesions undergo ulceration, necrosis, and epithelial hyperplasia. Healing occurs sequentially with inflammation, proliferation, and remodeling.

What differentiates the cutaneous lesions of MPVX from SPX is that in SPX, asynchronous lesions such as papules, vesicles, and crusts might all be seen at the same time [76]. Around 10% of monkeypox patients might present with ocular lesions. Almost 25% of confirmed patients reported conjunctivitis in the DRC [77]. Bacterial superinfection (8-30% of cases) might cause permanent pitted scarring in the long run. Sometimes lesions may be present on the conjunctiva and cornea. The skin lesions are highly correlated with periods of transmissibility. H-MPVX cases might have complications such as abscess formation, ulcerated and necrotic lesions, nausea and vomiting, diarrhea, encephalitis, bronchopneumonia, and airway compromise from lymphadenitis leading to hospitalization [65].

Very little information exists about the lab parameters in H-MPVX cases. Few studies have reported elevated blood urea nitrogen, leukopenia, thrombocytopenia, hypoalbuminemia, and raised hepatic transaminases [78,79].

Infection of index cases primarily occurs by direct contact with an infected animal's blood, skin, and lesions and bites from rodents. The zoonotic nature of the virus has been long confirmed across outbreaks, and it was associated with low human-to-human transmission. During the 1980s, 72.5% of infections in the DRC possibly had an animal source, and 27.5% of cases had a human source. Later in the 1990s, however, almost 88% of cases confirmed contact with an infected individual within the last 7-21 days. In the Nigerian outbreak of 2017 also, an obvious animal link could not be established, with almost 78% of cases giving a history of contact with an individual having monkeypox-like lesions. Rest reported contact with animals [47,60]. It is now known that secondary human transmission may occur by close contact with infected respiratory tract secretions, skin lesions of cases, or fomites contaminated with secretions and especially sustained face-to-face contact with patients. Patients become infected from the moment symptoms start until the scabs fall off. Congenital monkeypox with transmission from the mother to the fetus is also possible.

The current outbreak is novel regarding clinical manifestations and modes of transmission. We know that the current outbreak is milder, with hardly any fatalities, most likely because of the West African clade. Rash, pruritis, fever, and lymphadenopathy remain the hallmark of the disease. However, with an increase in the sexually transmitted disease burden, rash in the genital area (75%) is the most pronounced atypicality of the outbreak of 2022, in contrast to African patients, where it has been found in only 30% of patients. At this time, many patients present with a single lesion or a few lesions, sometimes localized around a specific area [65,80]. Lesions are asynchronous and pleomorphic, unlike the synchronous and monomorphic nature of lesions observed in earlier infections. More than 50% of patients currently present with 100 or more lesions [81].

Ulcerating genital lesions are a hallmark, another peculiarity of the current outbreak. Some cases in 2022 present with a single genital ulcer [1,2]. The genital lesions mostly precede the pustular rash, secondary disseminated infection, and satellite lesions in other body areas. MPVX at this time usually coexists with sexually transmitted infections (STIs), which makes diagnosis difficult. A thorough evaluation of genital lesions and rashes is a must, as STIs and MPVX may mimic each other [82]. The current outbreak shows new symptoms like proctitis, rectal pain, penile edema, central nervous system involvement, and pharyngitis with fewer skin lesions [83,84].

The possibility of coinfections (HIV, syphilis, and other STIs) and how they may impact the clinical course of the disease have recently been considered [3]. Reports emanating from African outbreaks have given variable results. A study reported 118 H-MPVX cases in which there were seven deaths. Four of these fatalities were in HIV patients [85,86]. It is expected that advanced HIV infection might be causative of severe MPVX disease and even mortality [87]. No specific recommendations are there for managing MPVX-infected HIV patients, except for close monitoring of CD4 counts. Non-replicating SPX vaccines like Imvanex (Bavarian Nordic) might be used for pre-exposure or post-exposure prophylaxis [88,89].

Superimposed bacterial infections over skin lesions are possible, especially in elevated IL-10 levels that potentiate inflammation and impact dermal regeneration and healing in H-MPVX cases. Cellulitis and sepsis might occur in patients with bacterial superinfections. Patients might be at risk of airway compromise due to pharyngeal edema.

A prospective cross-sectional study of MPVX cases in 2022 in Spain reported even more contrasting features, with lesions starting as homogenous papules rather than pustules and the presence of MPVX whitlows. SPX vaccination status did not have an impact on the severity [90].

The usual transmission mode through respiratory droplets, fomites, or close face-to-face contact, agreed upon across the decades, has now changed to primarily sexual transmission, specifically in MSM [91]. A study by Català et al. in Spain has concluded that MSM are highly prone to H-MPVX infection [90]. In the same study, it found that 71% of patients had an STI. Tightly interlinked sexual networks, close physical contact, and high-risk sexual behavior might be a reason for the easy spread of the virus in the gay, MSM, and bisexual communities witnessed in the current outbreak. while close physical contact, even without sex, can transmit infection, how exactly the virus spreads, even among MSM is, as of now, not so clear. Super spreader events like the festivals on Grand Canary Island in Spain or Belgium could be evaluated for better answers (Figure 3).

In an observational analysis of 54 patients with confirmed MPVX infection attending sexual health clinics in London between May 14 and 25, 2022, it was observed that 100% of the patients were MSM and had a median age of 41 years. Almost 24% lived with HIV, and 18% had no prodromal symptoms. They all presented with skin lesions, primarily anogenital (94%) and some oropharyngeal (7%). Cellulitis was present in 11% of the patients. Lymphadenopathy was present in 55% of the cases, and 25% had a concurrent STI [92]. A strikingly similar pattern was observed in 301 PCR-confirmed H-MPVX patients in Germany [93].

An Italian study of four MPVX cases among MSM, who had indulged in condomless intercourse, reported that all the patients were in good clinical condition and had no need for antiviral drugs. Their seminal fluid, genital and rectal lesions, feces, and saliva were positive for MPVX viral DNA [94]. Studies have also reported the presence of a rash exclusively at the site of close contact [95]. Conjunctivitis, edema of the eyelids, photophobia, corneal infections and scarring, keratitis, and blepharitis, have been reported in H-MPVX cases [96].

Diagnosis of monkeypox

Patients with new onset febrile illness, lymphadenopathy, and rash should raise suspicion on H- MPVX in the current scenario. A polymerase chain reaction (PCR) testing skin lesions/fluid, preferably from two separate lesions on different body parts, is used to confirm the diagnosis [97]. Testing for MPVX cannot be avoided in those with STIs, especially in MSM with risky sexual behavior; it should be a priority considering the high rate of concurrent STIs in the current outbreak. Symptoms of syphilis (fever, headaches, lymphadenopathy) might mimic monkeypox closely and confuse the physician. Lesion swabs, blood, and semen samples for PCR testing to identify MPVX should, therefore, form a part of the research protocol in suspected patients whether or not they have an STI. Serological testing might aid epidemiological investigations in understanding late clinical manifestations like encephalitis.

Drugs, vaccines, and preventive measures against MPVX

Anyone presenting with a fever and pustular rash and giving a history of travel to an endemic region should be screened for MPVX. Confirmed patients should immediately be isolated until lesions resolve (crusts formed or scabs fallen off); till then, lesions could be kept covered to prevent spread. The CDC or local government hospitals and authorities should promptly start contact tracing.

Drugs

There is a lack of evidence-based guidelines for treating monkeypox. Management is usually supportive. Recently the WHO has come up with interim guidelines for clinical management (Table 2) [81].

**Table 2:** Symptomatic treatment in H-MPVX cases IL: interleukin; PPIs: proton pump inhibitors; H-MPVX: human monkeypox infection Source: Clinical management and infection prevention and control for monkeypox: Interim rapid response guidance, 10 June 2022 [95]

Sign/Symptom	Treatment
Fever	Analgesics, antipyretics, cool compresses
Difficulty in breathing, bronchoconstriction, hypoxia, cough	Chest physiotherapy, nebulizers, noninvasive ventilation, bronchoscopy, antibiotics, bronchodilators
Infection, sepsis	Antibiotics, steroids, insulin, hemodynamic support with vasopressors, fluids, supplemental oxygen
Bacterial superinfection on skin	Antibiotics, wound care, negative pressure treatment, incision drainage
Ocular disease	Analgesics, ophthalmic antivirals, antibiotics and steroids, lubricants,
Local or systemic inflammation	Analgesics, steroids, antipyretics, IL antagonists
Lymphadenopathy	Analgesics, antipyretics, wet compresses
Skin lesions	Topical antibiotics, surgical debridement, disinfectant and lubricant dressings, washing the lesion with soap and water
Anxiety	Counseling, diazepam (moderate anxiety),
Agitation	Diazepam, haloperidol
Diarrhoea and vomiting	Antidiarrhoeals, fluids, anti-emetics
Dyspepsia	Proton pump inhibitors
Wound healing	Antibacterial ointment, vitamin C, moisturized dressing
Pain	Mild- paracetamol severe- tramadol, morphine
Allergy, pruritis	Antihistaminics

Cidofovir, brincidofovir, and tecovirimat possess antiviral activity against MPVX (Table 3).

**Table 3:** Treatment, dose, route, and duration of antivirals for H-MPVX PO: per oral; IV: intravenous; L: liter; CMV: cytomegalo virus; H-MPVX: human monkeypox infection Source: Clinical management and infection prevention and control for monkeypox: Interim rapid response guidance, 10 June 2022 [81]

Age group	Variables	Tecovirimat	Brincidofovir	Cidofovir
Adults	Route (Dose)	Oral: (600mg PO every 12 hours) Intravenous: (To be administered over 6 hours) 3 kg to < 35 kg: 6 mg/kg every 12 hours; 35 kg to < 120 kg: 200 mg every 12 hours; > 120 kg: 300 mg every 12 hours	Oral: < 10 kg: 6 mg/kg; 10–48 kg: 4 mg/kg; > 48 kg: 200 mg (20 mL)	Intravenous: 5 mg/kg IV once weekly; Must be given with oral probenecid: 2 grams 3 hours prior to each dose and 1 gram at 2 and 8 hours after completion of the infusion; Must be given with at least 1 L of 0.9% normal saline over a 1-2-hour period before each infusion
	Duration	14 days	Once weekly for 2 doses, on days 1 and 8	Once weekly × 2 weeks, then once every other week (Based on treatment for CMV retinitis)
Pediatrics	Route (Dose)	Oral: 13–25 kg: 200 mg every 12 hours; 25–40 kg: 400 mg every 12 hours; > 40 kg: 600 mg every 12 hours Intravenous: (Must be given over 6 hours) 3–35 kg: 6 mg/kg every 12 hours; 35–120 kg: 200 mg every 12 hours; > 120 kg: 300 mg every 12 hours	Oral: < 10 kg: 6 mg/kg; 10–48 kg: 4 mg/kg; > 48 kg: 200 mg (20 mL)	Intravenous: 5 mg/kg IV once weekly; Must be given with oral probenecid: 2 grams 3 hours prior to each dose and 1 gram at 2 and 8 hours after completion of the infusion; Must be given with at least 1 L of 0.9% normal saline over a 1–2-hour period prior to each infusion.
	Duration	14 days	Once weekly for 2 doses, on days 1 and 8	Once weekly × 2 weeks, then once every other week (Based on treatment for CMV retinitis)

Cidofovir is the only FDA-approved drug for CMV retinitis. In MPVX infection, it works by preventing virus replication by inhibiting DNA polymerase. Reported side effects of cidofovir are nausea, alopecia, myalgia, fever, metabolic acidosis, neutropenia, decreased intra ocular pressure (IOP), uveitis, and iritis. Cidofovir was used on a 28-month-old boy with atopic dermatitis who developed severe eczema vaccinatum after he came in contact with his father, who had received an SPX vaccination [98]. Cidofovir-associated electrolyte abnormalities and renal toxicity have also been reported [99]. Cidofovir is a pregnancy class C drug.

Brincidofovir was approved for the treatment of SPX in 2021. It possesses lesser renal toxicity in contrast to cidofovir. Brincidofovir is available as an oral tablet or suspension administered to patients in two doses one week apart [100]. Reported side effects of brincidofovir include nausea, vomiting, diarrhea, abdominal pain, and raised hepatic transaminases. It is contraindicated in pregnancy because of a chance of teratogenicity. In fact, patients on the drug should use proper precautions and avoid becoming pregnant [55]. We have little data on the efficacy of cidofovir and brincidofovir in human MPVX; activity against poxviruses has been demonstrated in in vitro and animal studies [101,102]. In May 2022, three patients with H-MPVX were treated with brincidofovir. However, the drug was stopped in all three patients due to elevated liver enzymes, a characteristic side effect of the antiviral [55].

Tecovirimat is the first antiviral drug approved under the FDA Animal Rule for treating SPX in patients weighing at least 3kg. Several in vitro, preclinical and clinical trials have demonstrated the efficacy and safety of tecovirimat against orthopoxviruses SPX, vaccinia, MPVX, cowpox, camelpox, and mousepox viruses. In the context of MPVX, it seems to possess activity against the V061 gene that encodes for p37, an integral envelope protein essential for extracellular virus formation. ST-246 prevented the formation of plaques and virus-mediated cytopathic effects in cell culture. BALB/c mice, pretreated with oral ST-246, were protected from lethal infection with the vaccinia virus after nasal inoculation. Mice treated with ST-246 survived an infection, acquired protective immunity, and were resistant to a new lethal dose of vaccinia virus [103,104]. Mild to moderate hypoglycemia was observed in a Phase 1 clinical trial of the drug when co-administered with repaglinide. Human volunteers could tolerate the drug adequately when administered for 14 days [31]. There is a single case report of a British patient given tecovirimat in 2021. He experienced a short duration of disease and decreased viral excretion. Blood and upper respiratory tract became PCR negative after 48 hours of starting the drug and remained PCR negative at 72 hours. Haematological, renal, and liver profiles remained stable, and he was sent home after completing therapy [55]. The side effect profile of tecovirimat in an expanded safety trial of 359 human volunteers was similar to the placebo. The side effects observed were headache, nausea, and abdominal pain. With the approval of Tecovirimat, bioterrorism preparedness has attained a new height. Currently, it is stockpiled by the United States Strategic National Stockpile and may be released in exceptional circumstances [105].

Tecovirimat may be considered in H-MPVX cases who have severe disease (e.g., hemorrhagic disease, confluent lesions, sepsis, encephalitis, or other conditions requiring hospitalization) or are at high risk of severe disease (immunocompromised, pediatric population, pregnant or breastfeeding, people with a history of atopic dermatitis, people with other complications including gastroenteritis, bacterial skin infection, etc.). It may also be considered in patients with aberrant diseases such as accidental implantation in the eyes, mouth, genitals, or anus. Tecovirimat is contraindicated in those who have a known allergy to it or are unable to give consent. It is available as an oral capsule (200 mg) and an IV formulation. IV formulation is not to be used in patients with CrCl <30mL/min or in children less than two years of age, given their immature renal tubular function. Absorption is better with a full fatty meal. Headache, nausea, vomiting, dizziness, upper abdominal pain with oral formulations and infusion site reactions, swelling, and erythema with IV formulations can be expected as side effects. Hypoglycemia with repaglinide and therapeutic failure of midazolam has been reported as drug interactions with Tecovirimat. Drug interactions have also been reported with omeprazole, bupropion, atorvastatin, maraviroc, tadalafil, voriconazole, and tacrolimus. The drug is available free of cost under the Expanded Access Investigational New Drug (EA-IND). Tecovirimat can be requested from the CDC for patients with suspected, probable, or confirmed MPVX state/territorial health department or CDC through the CDC Emergency Operations Center (770-488-7100). GNH India Pharmaceuticals Limited (Mumbai, India) is a WHO Good Distribution Practices (GDP)-certified supplier and ISO 9001 2015-certified pharmaceutical importer of tecovirimat in India, which has entered into a licensing agreement with the innovator company of tecovirimat i.e SIGA Technologies, Inc. (New York City, United States), through which tecovirimat can be procured in India. However, all said and done, the complete picture of tecovirimat in humans is yet to be uncovered, and strict caution needs to be exercised when contemplating its usage [106].

In severe cases, a combination of tecovirimat with brincidofovir might be used. All these antivirals are only available under EA-IND protocol. NIOCH-14 antiviral drug therapy and its effectiveness in H-MPVX is another gap in knowledge that needs to be covered. FDA approves vaccinia immunoglobulin (VIG) for specific complications of vaccinia vaccination (eczema vaccinatum, generalized vaccinia), but data regarding the utility of VIG in MPVX and SPX is limited. VIG may be given to exposed persons, and H-MPVX and SPX patients with severe immunodeficiency as vaccination with vaccinia virus are contraindicated in them [106].

Vaccines

The initial vaccines for SPX were made from bovine SPX pustules. Later attenuated strains of SPX were used for mass immunization, leading to the eradication of SPX. The vaccine’s effectiveness slowly waned over an individual’s lifespan; however, protection against severe disease was present throughout life. These vaccines came with inherent toxicity. The inoculation lesion sometimes extended locally, leading to a fatal gangrenous lesion. A self-limiting generalized extension of the lesion was another complication. Encephalomyelitis, focal aphasia, and hemiplegia in children under two were also reported [13.14]. Another more frequent complication is eczema vaccinatum, which occurs in those with eczema who experience skin-to-skin contact with someone who has had an SPX vaccine. Although SPX is eradicated, vaccines might still be given to those working in the military or a research facility devoted to SPX testing. The SPX vaccine offers cross-protective community immunity against MPVX because of the considerable nucleotide identity (96.3%) in the center of the two viruses [107].

Currently, three vaccines might work towards preventing and attenuating monkeypox symptoms. An attenuated non-replicating live viral vaccine (JYNNEOS, Invanex, IMVAMUNE®) adapted from the modified vaccinia Ankara-Bavarian Nordic was approved for SPX and MPVX in adults above 18 years at high risk for SPX or MPVX by the European Medicines Agency (EMA) in 2013 and in the United States in 2019. The vaccine is currently a part of the Strategic National Stockpile. JYNNEOS might be used for susceptible people below 40 years of age rather than older individuals who most likely possess cross-protective immunity against MPVX. It is given subcutaneously in two doses, 28 days apart [106].

ACAM2000 is another live vaccinia virus that was approved by FDA in 2007 for people at high risk for SPX disease. The CDC can allow the use of ACAM2000 in case of an *Orthopoxvirus* outbreak as per an EA-IND protocol. It is given percutaneously in a single dose using a bifurcated needle by multiple puncture technique [106].

A replication-competent vaccine, Aventis Pasteur Smallpox Vaccine (APSV), may be used under EA-IND for SPX prevention if the licensed vaccines are unavailable or contraindicated [106].

Replication-competent vaccines seem less preferred as they may lead to severe cutaneous manifestations, eczema vaccinatum, and progressive vaccinia in immunocompromised and atopic individuals. With the current outbreak witnessing more infections in people having STIs, it is advisable to prefer JYNNEOS [108].

In the United States MPVX outbreak of 2003, where approximately a dozen cases were identified, the SPX vaccine was deployed as pre-exposure and post-exposure prophylaxis for some contacts of MPVX cases [68]. Vaccine administration early enough can lead to attenuation of and even complete protection from symptoms of monkeypox. Vaccinia immunoglobulin may be used for postexposure prophylaxis where the SPX vaccine is contraindicated.

Pre-Exposure Prophylaxis Against MPVX

Pre-exposure vaccination is recommended for research personnel and diagnostic and clinical response teams connected with *Orthopoxvirus* research and management. In addition, healthcare professionals that administer ACAM2000 may also be vaccinated.

Preventive Measures in Contacts of H-MPVX Cases and Suspects

MPVX usually spreads through droplets, fomites, or direct contact. Infected individuals should immediately start wearing masks, cover their lesions, and stay isolated until lesions scab and fall off and dermal reepithelialization begins. Additionally, the CDC has not ruled out the possibility of airborne transmission and advises airborne infection control measures wherever applicable.; including wearing an N-95 mask or personal protective equipment (PPE) by caregivers and healthcare professionals taking care of cases and suspects of H-MPVX. Contacts are usually classified as close (in the same room within1m distance and without PPE with a case or suspect of H-MPVX), direct (contact with clothing, fomites, linen used by a confirmed case or suspect without PPE), and low-risk (those not meeting above criteria). Contacts of a suspected or confirmed case of H-MPVX should be identified actively. They must be made aware of the symptoms of monkeypox and asked to restrict social interactions and monitor temperature twice daily for at least 21 days. Isolation in negative pressure rooms is advised. Suppose any contacts present with fever or other signs and symptoms of MPVX; self-isolation and contacting the authorities for further testing and guidance are recommended. If possible, the linen of infected persons and suspects should be washed in hot water and bleach. Direct contact with fomites, clothing, and bedding should be avoided. Linen should not be handled in a manner that might disperse infectious particles. Dishes should not be shared and washed in soap and water. Fomites and surfaces can be disinfected with a hospital-grade disinfectant or a 1:100 dilution of household sodium hypochlorite (bleach).

The new age SPX vaccine JYNNEOS (Bavarian Nordic) is FDA approved for the prevention of monkeypox. Vaccination of close contacts has given suitable containment in previous outbreaks [109-112].

Post-Exposure Prophylaxis Against MPVX

Since we do not have many effective drugs for MPVX, immediate vaccination can be pivotal for community health protection once a diagnosis has been confirmed. Post-exposure vaccination within four days and monitoring are recommended for high-risk exposures. In intermediate degrees of exposure, vaccination decision depends on an individualized benefit-risk assessment. In low or uncertain exposures, simple monitoring may suffice without vaccination.

There are multiple factors responsible for the resurgence of MPVX, and it is a cause for global concern (Table 4).

**Table 4:** Reasons for the resurgence of MPVX MPVX: monkeypox virus; SPX: smallpox virus; R0: basic reproduction number

Possible reasons
Waning Orthopoxvirus immunity due to cessation of SPX vaccination.
Increasing R_0_ (Basic reproduction number) of the virus after eradication of SPX.
Possible viral genome mutation (a gene loss that might increase human-to-human transmission)
Improved affinity and tolerability for the human host
Chances of a super spreader event that might have caused an explosion of cases
Major population growth
Increased global connectivity
Deforestation and changes in land usage patterns

As a result of these factors, there is a fear of whether the virus is going to take similar proportions as COVID-19. However, It is unlikely that something like this could happen for the following reasons. Unlike COVID- 19, airborne transmission has not yet been found to be responsible for transmission in MPVX. Tecovirimat is a drug that was specifically designed for SPX and might show equivalent efficacy in MPVX, considering both are orthopoxviruses. This is unlike COVID-19, for which, at best, we have repurposed drugs. We do not need to wait for MPVX vaccine development, as we already have several vaccines being used in various settings; pre-exposure, post-exposure prophylaxis, and even for treatment in MPVX. Although a severe problem might present in the future if the MPVX virus with an excellent ability to spread, mutates and reaches a population with no immunity against it.

Based on research and evidence being generated globally, there are certain recommendations that should be considered (Table 5).

**Table 5:** Recommendations based on the reviewed data about the past and the ongoing outbreak MPVX: monkeypox virus; H- MPVX: human-monkeypox virus; HCP: healthcare personnel; PPE: personal protective equipment; MSM: men who have sex with men; STIs: sexually transmitted infections; R0: basic reproduction number

Conclusions	Recommendations
Information about previous MPVX outbreaks and the current outbreak is lacking.	International collaboration and studies exploring the epidemiology and risk factors of the disease are vital for optimising public health protocols for tackling the pandemic. HCP training about effective diagnostic, therapeutic and preventive protocols should be initiated. Social media campaigns to make the general public aware of all aspects of H- MPVX, including identification, isolation, information, prevention, and treatment of H-MPVX infection.
Randomised controlled trials assessing the effectiveness of Cidofovir, Brincidofovir and Tecovirimat and other antivirals in H- MPVX are lacking.	Well-controlled studies or observational studies of Cidofovir, Brincidofovir and Tecovirimat in H- MPVX need to be planned.
The clinical picture is changing a.A milder form of disease b.Pleomorphic lesions c.Asynchronous lesions d. More frequent genital lesions and rash e. Fewer skin lesions than before f. Presence of ulcerating genital lesions/single lesion g. New symptoms- rectal pain, proctitis	Observational studies and case reports should be published. Unusual signs and symptoms should be kept in mind by physicians A high index of suspicion is to be kept in any patient who presents with features suggestive of MPVX and any other hitherto unknown sign. Early recognition and timely intervention are the keys to success
Orthopoxvirus-mediated immunity is waning, and the R_0_ of the virus has increased. New and evolved outbreaks may happen in future.	Vaccination is going to be vital in the control of future outbreaks. Ring vaccination and vaccinating contacts of the index case and high-risk groups can curtail the spread of the disease. Mass immunisation might be an option in areas where MPVX becomes endemic.
Vulnerable groups- children, pregnant and immunocompromised may have severe infections and bad outcomes.	Children with H-MPVX infection should be kept under close surveillance in their homes or preferably in hospitals. Drug therapy might be considered. A high index of suspicion in pregnant females with a rash and lymphadenopathy is to be maintained. Further epidemiological investigations about the complete picture of the disease in vulnerable groups need to be conducted.
The long incubation period of the virus might be a problem for HCPS and caregivers.	Appropriate information about masks, PPE, isolation, disinfection of fomites, bed linen and clothes should be provided to people who might come in contact with confirmed or suspected cases of MPVX.
The virus has spread globally.	Travel at this time could be avoided completely, or at least to locations with reported cases. Factors facilitating this massive multi-country transmission as of now need to be explored and addressed. Efforts should be taken to contain the virus through identification, isolation, information, contact tracing, ring vaccination, post-exposure vaccination and mass immunisation if need be.
The virus is currently mostly clustering around the MSM community.	A strongly worded message avoiding high-risk sexual behaviour needs to be sent out there. MSM suspecting MPVX infection need to be able to visit a physician immediately without fear of social alienation The potential for H- MPVX cases being confused with STIs must be kept in mind. Active Tracking for Monkeypox as a communicable disease can be started, similar to what is being done for COVID-19. Studies should be planned to explore how exactly the virus is travelling, especially in these communities.
The sequenced viral genome of the current outbreak closely resembles the West African clade with low mortality of about 1%. However, different studies have different genome sequencing reports	Genome sequencing from both the primary and the disseminated rash is essential to understand the evolution and adaptations of the virus during the current outbreak.
A “misinfodemic” with several myths and doubts about MPVX is creating panic.	Social media campaigns providing the correct information about MPVX prevention and control will help not only allay the panic but serve as an important control strategy.
Little data exists about lab abnormalities seen in H- MPVX cases.	More focus needs to be given to the collection of such data through well-planned observational studies.
Animal sources will continue to be a source of zoonotic infection	Veterinarians have an important role to play in the current outbreak. Screening of animals especially coming from endemic regions, is vital to prevent zoonotic transmission.

## Conclusions

There are many questions and few answers, about monkeypox and its impact on humanity. Consistent efforts should be taken to characterize the disease through well-conducted studies so that effective management protocols can be designed and countries are better prepared to tackle new and more serious outbreaks. High-risk populations need to be prioritized. Specifically, directed campaigns for MSM can be most helpful at this time. The factors responsible for global spread need to be minutely understood and appropriate measures taken to control any chance of the disease taking the form of a pandemic. Population-level education awareness programs to make the masses aware of crucial facets of the disease (prevention, clinical features, and management) can help to ameliorate the disease and its associated morbidity. Targeted training of healthcare personnel to identify the disease as early as possible is vital, especially in changing the clinical picture and transmission routes.
